# Clinical effects of polynucleotide with hyaluronic acid intradermal injections on facial erythema: Effective redness treatment using polynucleotides

**DOI:** 10.1111/srt.70034

**Published:** 2024-09-11

**Authors:** Dongkeun Kenneth Lee, Myungjune Oh, Michael James Kim, Seung Min Oh

**Affiliations:** ^1^ O.N Clinic Dermatology & Plastic Surgery Seoul Republic of Korea; ^2^ Aeon Medical and Aesthetic Centre Singapore Singapore

Dear Editor

There have been previous publications reporting on the effects of polynucleotides (PN) in treating facial erythema. PN are biocompatible substances derived from fish germ cells^1^ and have been utilized in aesthetic clinics for many years.^2^ PN are especially notable among many other biostimulators because of their distinct scaffold structures^3^ and beneficial effects in improving overall skin conditions, such as pore size, skin tone, skin thickness, fine wrinkles, and sagging.^4^ In a study conducted by Lee et al. a large‐scale survey was conducted among Korean aesthetic physicians who incorporate PN in their practice. The survey revealed that a significant number of aesthetic physicians, based on their clinical experiences, agreed on PN's beneficial effects in reducing redness and stabilizing vascular conditions.^5^ Following the findings from these studies, we conducted a small‐scale retrospective case study as a proof of concept'' before initiating a clinical trial to investigate PN's effectiveness in treating erythema. Here, we present case reports of two female patients with generalized facial erythema.

Amongst patients who visited the clinic to have PN injection procedures with concerns of skin dehydration, erythema, and signs of aging; those who received treatments (including laser treatments, topical application, or skin care programs) other than PN which can affect results in their erythema, were first excluded to remove any confounding factors. Two Korean female patients were selected based on similar ages (41 and 45) and the completion of three sessions of PN injections at 2 week intervals. They re‐visited the clinic at 2 weeks (W6) and 4 weeks (W8) after the completion of treatments to assess the effects of PN injections (Figure 1). 2 cc of PN (Rejuran; PharmaResearch Products Co., Ltd.; Seoul,Republic of Korea) was mixed with 2 cc of non‐crosslinked hyaluronic acid (New‐Arti; PharmaResearch Products Co., Ltd.; Seoul, Republic of Korea) and 0.5 cc of lidocaine (Huons**;** Jecheon‐si, Chungcheongbuk‐do, Republic of Korea). The use of a mixture of PN and non‐crosslinking HA is common due to its well‐known efficacy.^6^ Lidocaine was added to reduce pain during injection.

**FIGURE 1:**
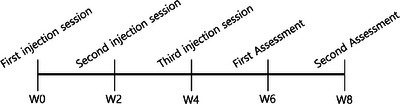
Injection procedure schedule. Two patients visited the clinics for PN injection procedures following the above schedule. PN, polynucleotides.

For anesthesia, topical lidocaine cream (Lidocan Cream; DaehanNupharm Co.,Ltd; Gyeonggi, Republic of Korea) was applied to both cheeks, chin, and the forehead for 30 min. After removing the cream, the treatment areas were disinfected with 5% chlorhexidine. Cold CO_2_ medical sterilized gas (Cryoblast; Chungwoo Medical; Seoul, Republic of Korea) was sprayed on the face until the temperature of skin reached 4–5°C to further minimize pain. Patients received PN‐mixture injections intradermally using a serial puncture technique. 2 cc on the cheeks/chin and 0.5 cc on the forehead were injected in a grid‐like pattern, with each injection site placed about 0.5 cm from each other.

Consent for assessment was taken after the 3rd injection was completed and before 2 weeks passed from the final injection. The clinical efficacy was measured based on self‐assessment using the Global Aesthetic Improvement Scale (GAIS) reported at 2 weeks (W6) and 4 weeks (W8) after the completion of the treatment series. The physician (DKL) who conducted the injection procedures evaluated changes in erythema as well using Clinician's Erythema Assessment (CEA) based on the patients' photographs taken at every visit (Table 1).

**TABLE 1:** GAIS scoring and CEA.

GAIS		CEA	
Score	Grade	Score	CEA
3	Very much improved	0 = Clear	Clear skin with no sign of erythema
2	Much improved	1 = Almost clear	Almost clear; slight redness
1	Improved	2 = Mild	Mild erythema, definite redness
0	No change	3 = Moderate	Moderate erythema; marked redness
–1	Worse	4 = Severe	Severe erythema; fiery redness

*Note*: Two patients reported self‐assessment on W6 and W8 based on five‐scale grading system of GAIS scores. The physician evaluated photographs taken on W0, W2, W4, W6, and W8 of patients' visit to the clinic and scored changes in erythema based on five‐scale grading system of CEA.

Abbreviations: CEA, clinician's erythema assessment; GAIS, global aesthetic improvement scale.

Self‐assessment using GAIS score was reported at 2 weeks (W6) and 4 weeks (W8) after the completion of the series of three injection procedures. Overall, there were increases in both of the patients' satisfaction throughout the repeated sessions of PN injections. In Case 1, the GAIS scores on W6 and W8 were 1 and 2, respectively. In Case 2, the GAIS score was 1 and 3, respectively at the same assessment points.

The physician retrospectively evaluated changes in facial erythema using CEA scores based on the patients' photographs taken on W0, W2, W4, W6, and W8. In case 1, CEA scores were 3, 3, 2, 1, and 1, respectively for those periods. In Case 2, CEA scores were 4, 3, 3, 2, and 1, respectively.

The clinical before‐after photos are as follow (Figure 2).

**FIGURE 2:**
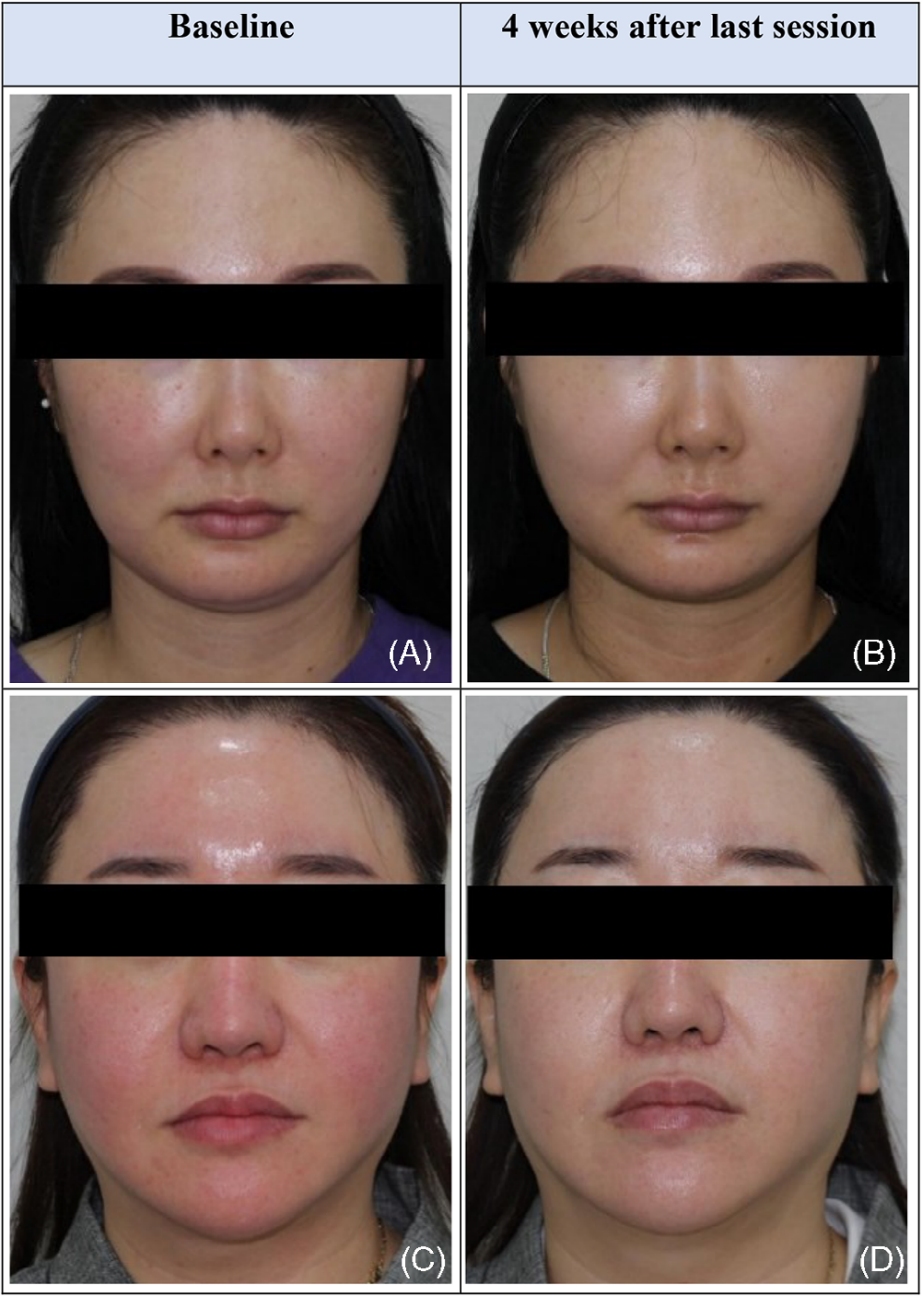
Improvement of facial erythema after three sessions of PN injections. Patients photographed on week 0 (A, C) and 4 weeks after the third injection session (B, D). Improvement in erythema on the forehead (D), cheeks and chin can be seen (B, D). PN, polynucleotides.

Previous studies investigated the skin rejuvenation effects of PN for different parameters of skin conditions such as skin tone, elasticity, fine wrinkles, hydration, and pore tightness. From the survey conducted to over 500 physicians practicing aesthetic medicine in South Korea, there was significant acceptance and empirical evidence that PN are also effective in improving facial erythema.^5^ The results from this small‐scale retrospective case study supports the before mentioned survey results and suggests that PN are indeed clinically effective in facial erythema treatment. The anti‐inflammatory properties^7^ of PN, coupled with its role in *de novo* DNA synthesis via the salvage pathway,^8^ likely contribute to its therapeutic effects on erythema. These promising results warrant further investigation through controlled clinical trials to establish standardized treatment protocols.

We appreciate your time and consideration in reviewing our manuscript. Thank you for the opportunity to share our findings with the scientific community.

And we thank the patients who consented to the retrospective analysis.

## CONFLICT OF INTEREST STATEMENT

S.M.O. and M.J.K. are advisory members of PharmaResearch Products Co., Ltd.

## Data Availability

The data that support the findings of this study are available from the corresponding author upon reasonable request.
